# Expressional alterations in functional ultra-conserved non-coding rnas in response to *all*-*trans* retinoic acid - induced differentiation in neuroblastoma cells

**DOI:** 10.1186/1471-2407-13-184

**Published:** 2013-04-08

**Authors:** Karen M Watters, Kenneth Bryan, Niamh H Foley, Maria Meehan, Raymond L Stallings

**Affiliations:** 1 Cancer Genetics, Department of Molecular & Cellular Therapeutics, Royal College of Surgeons in Ireland, Dublin, Ireland; 2 Children’s Research Centre, Our Lady’s Children’s Hospital Crumlin, Dublin, Ireland

**Keywords:** ATRA, neuroblastoma, Transcribed ultra-conserved regions, Differentiation

## Abstract

**Background:**

Ultra-conserved regions (UCRs) are segments of the genome (≥ 200 bp) that exhibit 100% DNA sequence conservation between human, mouse and rat. Transcribed UCRs (T-UCRs) have been shown to be differentially expressed in cancers versus normal tissue, indicating a possible role in carcinogenesis. All-trans-retinoic acid (ATRA) causes some neuroblastoma (NB) cell lines to undergo differentiation and leads to a significant decrease in the oncogenic transcription factor MYCN. Here, we examine the impact of ATRA treatment on T-UCR expression and investigate the biological significance of these changes.

**Methods:**

We designed a custom tiling microarray to profile the expression of 481 T-UCRs in sense and anti-sense orientation (962 potential transcripts) in untreated and ATRA-treated neuroblastoma cell lines (SH-SY5Y, SK-N-BE, LAN-5). Following identification of significantly differentially expressed T-UCRs, we carried out siRNA knockdown and gene expression microarray analysis to investigate putative functional roles for selected T-UCRs.

**Results:**

Following ATRA-induced differentiation, 32 T-UCRs were differentially expressed (16 up-regulated, 16 down-regulated) across all three cell lines. Further insight into the possible role of T-UC.300A, an independent transcript whose expression is down-regulated following ATRA was achieved by siRNA knockdown, resulting in the decreased viability and invasiveness of ATRA-responsive cell lines. Gene expression microarray analysis following knockdown of T-UC.300A revealed a number of genes whose expression was altered by changing T-UC.300A levels and that might play a role in the increased proliferation and invasion of NB cells prior to ATRA-treatment.

**Conclusions:**

Our results indicate that significant numbers of T-UCRs have altered expression levels in response to ATRA. While the precise roles that T-UCRs might play in cancer or in normal development are largely unknown and an important area for future study, our findings strongly indicate that the function of non-coding RNA T-UC.300A is connected with proliferation, invasion and the inhibition of differentiation of neuroblastoma cell lines prior to ATRA treatment.

## Background

Neuroblastoma (NB) is a highly heterogenous childhood cancer that arises from precursor cells of the sympathetic nervous system [1]. Clinical behaviour of these tumors, ranging from spontaneous regression to rapid progression and death due to disease, is highly correlated with a number of genomic alterations involving ploidy, MYCN amplification (MNA), and large-scale genomic imbalances such as loss of chromosome 1p, 3p, 11q and gain of 17q. MNA and loss of heterozygosity on chromosome 11q are particularly associated with aggressive disease course and represent independent genetic subtypes of NB. Each genetic subtype of NB, such as MNA or 11q-, also has significant differences in the expression patterns of large sets of protein coding genes [2-6], and in the expression profiles of non-coding RNAs such as microRNAs [7-11].

Ultra-conserved regions (UCRs) are by definition DNA segments that are at least 200 bp in length and that are 100% conserved between human, rat and mouse genomes. Four hundred and eighty-one such regions have been identified [12]. UCRs are comprised of three basic types – intragenic (39%), intronic (43%) and exonic (15%), which also includes ‘partly exonic’ and ‘exon containing’. Approximately 3% of UCRs are not easily classified, due to their juxtaposition with alternative splice variants of host genes and the resulting variable annotation. Calin *et al.*[13] carried out the first analysis of transcribed UCRs (T-UCRs) in cancer, demonstrating that approximately 9% of the 962 possible T-UCRs (sense + anti-sense) were aberrantly transcribed in either carcinomas or leukemias relative to normal tissue. Most significantly, the authors further demonstrated that siRNA-mediated down-regulation of one T-UCR (T-UC.73A) significantly increased apoptosis in a colorectal cancer cell line. Two recent studies have demonstrated that analysis of UCR expression signatures can also be applied to the evaluation of NB tumors [14,15]. Differential UCR expression profiles were shown to be associated with outcome in short-term versus long-term survivors with high-risk, stage 4 NB [15]. In addition, Mestdagh *et al*., found an expression signature of up-regulated T-UCRs in MNA compared to non-MNA tumors [14].

The synthetic retinoic acid, 13-cis-retinoic acid, is an established component of the treatment given to children with high-risk NB to reduce minimal residual disease [16,17] and exposure of a number of NB cell lines, such as SK-N-BE, to ATRA induces neural cell differentiation along with down-regulation of MYCN [18]. Here, we identify T-UCRs that are responsive to the retinoid, all-trans-retinoic acid (ATRA), across three ATRA-sensitive cell lines and investigate the functional role of the deregulated transcript T-UC.300A.

Previous studies analyzing UCR expression in NB have used qPCR, involving reverse transcription with random primers, which is unable to distinguish between transcripts originating from the sense or the anti-sense genomic strand. Our approach involved the construction of tiling arrays for 962 UCR regions, allowing for the detection of both sense and anti-sense transcripts and for expression of host genes. We identified and validated a number of T-UCRs that are differentially expressed following ATRA-induced differentiation. Further insight into a functional role for T-UC.300A was achieved by siRNA knockdown resulting in the decreased viability and invasiveness of ATRA-responsive cell lines. As T-UC.300A is down-regulated following ATRA treatment, our findings strongly indicate that its function is connected with the increased proliferation and invasion of NB cells prior to ATRA-treatment.

## Methods

### Cell culture

The NB cell lines SK-N-BE and SH-SY5Y were obtained from the American Type Culture Collection (ATCC). Cell culture medium consisted of Ham’s F12 and EMEM (50:50) supplemented with fetal bovine serum (10%), non-essential amino acids (0.5%), L-glutamine (0.5%) and penicillin/streptomycin (1%). The LAN-5 cell line was obtained from the Children’s Oncology Group Repository. LAN-5 culture medium consisted of RPMI supplemented with penicillin/streptomycin (1%). All cell culture reagents were obtained from GIBCO.

### ATRA treatment

All-trans retinoic acid (ATRA) was administered daily (5 μM final conc.) to cells over a period of 7 days. Treated and untreated cells were fixed using 4% paraformaldhyde (Sigma) and permeabilised in 0.5% Triton X-100. Cells were probed using the neuronal marker βIII Tubulin (Abcam), and subsequently were incubated with the flourescein-conjugated goat anti-rabbit Alexa Flour 488 antibody (Invitrogen). Cells were washed in PBS and then counterstained using DAPI. The Nikon TE2000s Fluorescence microscope was used to examine the cells and photographs were taken with the Hamamatsu (Orca 285) CCD Camera.

### Microarray design

The UCR custom tiling microarray was developed using Roche NimbleGens 4 × 72 K array, composed of four identical subarrays, tiling 962 ultra-conserved sequences (481 sequences in both sense and anti-sense orientation). Oligo lengths ranged from 50mer to 72mer, in order to maintain a similar Tm across all probes. The genomic coordinates of the 481 UCRs (Build HG17, May 2004) were obtained from http://users.soe.ucsc.edu/~jill/ultra.html and converted to Build HG18, using UCSC’s Batch Coordinate Conversion tool (http://genome.ucsc.edu/cgi-bin/hgLiftOver). In addition to the ultra-conserved sequences themselves, tiling coverage also spanned 2500 bases upstream and 500 bases downstream of the location of each UCR on both sense and anti-sense strands. Probes were designed in two separate containers (sense and anti-sense) on each subarray to facilitate independent data analysis. Array design is available in ArrayExpress– Accession number A-MEXP-1899.

For gene expression, the *Homo Sapiens* 4 × 72 K gene expression array from Roche NimbleGen was used.

### Sample preparation

The QIAGEN RNeasy Mini Kit (Cat. No. 74101) was used to extract RNA from cells (untreated at Day 0 and ATRA-treated at Day 7). DNase treatment was included to ensure complete removal of any genomic DNA that could affect results by also hybridising to the microarrays. RNA integrity was confirmed with the Agilent RNA Nano 6000 kit (Cat. No. 5067–1511) and an Agilent Bioanalyzer. Only RNA with an RNA Integrity Number (RIN) of >8 was used for microarray analysis. For tiling microarrays, double-stranded cDNA was synthesised from 3 ug total RNA using the ExpressArt TRinucleotide mRNA Amplification Micro Kit (AmpTec). *In vitro* transcription using the ExpressArt AminoAllyl Add-on Module (AmpTec) generated aminoallyl modified-anti-sense RNA (aRNA), which was subsequently incubated with NHS-Cy3 (Amersham). Following purification, 4 μg Cy3-aRNA was hybridised to microarrays.

For gene expression microarray analysis following siRNA knockdown of T-UC.300A or ATRA treatment, sample preparation was carried out as previously described [19].

### RT-PCR

Reverse transcription for transcribed UCRs was carried out on 1ug total RNA with gene-specific primers and the SuperScript III First-Strand Synthesis System for RT-PCR (Invitrogen) in a total reaction volume of 20 ul (T-UC.324: 5^′^ CCCCATCCCATATGACACTC 3^′^; T-UC.300A: 5^′^ AAAAGTGGAAATCAATTTTGAAGG 3^′^). Real-time PCR was carried out using custom designed TaqMan assays for T-UC.324 and T-UC.300A (Applied Biosystems).

### Western blot

Total protein was isolated from cells using a radioimmunoprecipitation assay (RIPA) lysis buffer (Sigma). Cell pellets were washed with PBS and solubilized in RIPA for 30 mins. Protein concentration was measured using the BCA assay from Pierce. Proteins were fractionized on 6% or 10% polyacrylamide gels, and blotted onto nitrocellulose membrane. MYCN protein and the neuronal marker β – III Tubulin were detected by Western Blot using the mouse monoclonal antibody SC-53993 (Santa Cruz) and the rabbit polyclonal antibody AB-8191 (Abcam) respectively.

### siRNA knockdown

siRNAs against T-UC.324 and T-UC.300A were designed using Dharmacons siRNA Design Centre. siRNAs were as follows:

T-UC.324: TTACCTAACCAGTGATTAA (sense strand sequence)

T-UC.300A: ATTCATGGATGGAGATTGA (sense strand sequence)

Cells were transfected with T-UCR siRNAs (final concentration 50 nM) or negative control siRNA (Dharmacon Negative Control #1, final concentration 50 nM) using the transfection reagent Lipofectamine (Invitrogen). Media was changed after 24 hrs. RNA was extracted 120 hrs after transfection.

### Acid phosphatase assay

Cells were transfected with siRNAs in 96-well plates using Lipofectamine, and plates were set up for timepoints 24-120 hrs. At each timepoint, the appropriate plate was washed twice with PBS. 10 mM *p*-nitrophenol phosphate in 0.1 M sodium acetate with 0.1% triton X-100 was added. Plates were incubated at 37°C for two hours and the reaction was stopped with 50 μL 1 M sodium hydroxide per well. Absorbance was measured at 405 nm using the Victor X3 Multi-Label Reader (Perkin Elmer).

### Cell proliferation assay

The effect of siRNA knockdown of T-UC.300A on the rate of cell proliferation in SH-SY5Y cells was assayed using the Cell Proliferation ELISA, BrdU (colorimetric) kit (Roche, Cat. No. 11 647 229 001). Cells were transfected with siRNA against T-UC.300A or a scrambled control and were cultured in a tissue culture grade, flat bottom 96-well plate for 96 hours (n=4). BrdU labeling solution was added to each well (final concentration 10 μM BrdU) and the cells were re-incubated for an additional 2 hours. The labeling medium was then removed and the cells were fixed and denatured. Denatured DNA was incubated with an anti-BrdU monoclonal antibody conjugated with peroxidase for 90 minutes, followed by washing. 100 μl/well substrate solution was added and the plate was incubated at +15 to +25°C for 5 minutes. Stop solution (25 μl 1M H_2_SO_4_) was added to each well and mixed thoroughly. Absorbance of samples was measured using the Victor X3 Multi-Label Reader (Perkin Elmer) at 450 nm, reference wavelength 690 nm. Following subtraction of the absorbance value for the blank control from all readings, the change in proliferation rate induced by knockdown of T-UC.300A was determined.

### Invasion assays

Cells were transfected in a 6-well plate. 48 hours after transfection, cells were trypsinized and counted. 2.5×10^4^ cells were added to BD BioCoat™ Growth Factor Reduced MATRIGEL™ Invasion Chambers (BD Biosciences) as per manufacturers’ instructions and incubated for a further 72 hrs. To determine the average number of invading cells, MATRIGEL inserts were stained with crystal violet and viewed under the microscope. The number of cells/field, in 5 random fields were counted at 200× magnification. Mean values of duplicate experiments were calculated and results subjected to t-test.

### Data processing and bioinformatics analysis

#### Gene expression arrays

The mRNA expression data was analysed using NimbleGens NimbleScan software version 2.4, which applied quantile normalization to the data [20] and expression values were obtained using the Robust Multi-Chip Average algorithm as described by Irizarry *et al*. [21]. Expressional alterations of 1.5 fold across both biological repeats were considered significant.

#### T-UCR tiling arrays

Microarrays were scanned using the GenePix 4000B scanner. Pair reports were generated using NimbleGen’s NimbleScan software version 2.4, and normalised by applying quantile normalisation. Median smoothing was first carried out on both the forward and reverse T-UCR tiling arrays with a window of 3 probes to remove outlying probe values. T-UCRs were considered expressed if their mean probe value was above the array background (median expression of the array). Only T-UCRs that were expressed in all 3 cell lines were considered. T-UCRs that were differentially expressed at least 1.5 fold in 2 out of 3 cell lines (n ≥ 4) were selected. For these selected T-UCRs, p-values were calculated across all 3 cell lines (n=6 biological samples) using Student’s t-test, and only T-UCRs with a p-value < 0.05 were considered significant.

#### Correlation of intragenic t-ucrs and host gene expression

Using the expression data from the ATRA-treated and untreated tiling arrays, intragenic T-UCRs transcribed in the same directions as their host genes were assessed for independent transcription relative to exonic expression, if exons were covered by the tiled region. Pearson’s correlation of T-UCR expression (mean probe value) and gene (mean expression of exonic probes) over experimental samples was used to assess T-UCR/Host gene expression correlation. Significance of this correlation was determined from a table of critical values for Pearson’s Correlation over various degrees of freedom.

#### Cluster analysis of intragenic t-ucrs expression

T-UCR expression was measured on the forward and reverse strand however this causes problems when calculating object similarity in cluster analyses i.e. every object will be represented twice in the expression dataset by two independent features sets, one on the forward strand and one on the reverse strand. The matrix was re-ordered by first re-orientating the arbitrary direction of T-UCR expression so that it was relative to the host gene with sense (S) referring to those T-UCRs transcribed with the host gene and anti-sense (A) referring to those T-UCRs transcribed in the opposite direction to the host gene. The S and A expressions for each T-UCR were then merged into a single feature set, thus the data matrix of 962 and *F* expression features was converted into a data matrix of 481 T-UCRS and *2F* features (*F*_*S*_*+ F*_*A*_*)*. Similarity of expression of intragenic T-UCRs was then determined using Spearman’s rank correlation. Hierarchical clustering was subsequently carried out using Ward’s metric and presented with accompanying heatmap visualization. Analysis was carried out using algorithms implemented in Java v1.6 and packages from R statistical programming language R version 2.8.0.

## Results

In order to examine the expression levels of all T-UCRs, a custom array was designed tiling across all UCRs on both plus and minus genomic strands. Thus, a potential 962 T-UCRs, transcribed from 481 genomic regions when taking into account forward and reverse transcripts, could be detected. Tiling also incorporated 2500 bases upstream and 500 bases downstream of each UCR. Discernment of the genomic strand from which each T-UCR originated was possible by synthesizing anti-sense RNA (aRNA), which is complementary to the original transcript, for Cy3 labelling and array hybridization.

### Differential expression of t-ucrs following atra treatment

**Figure 1:**
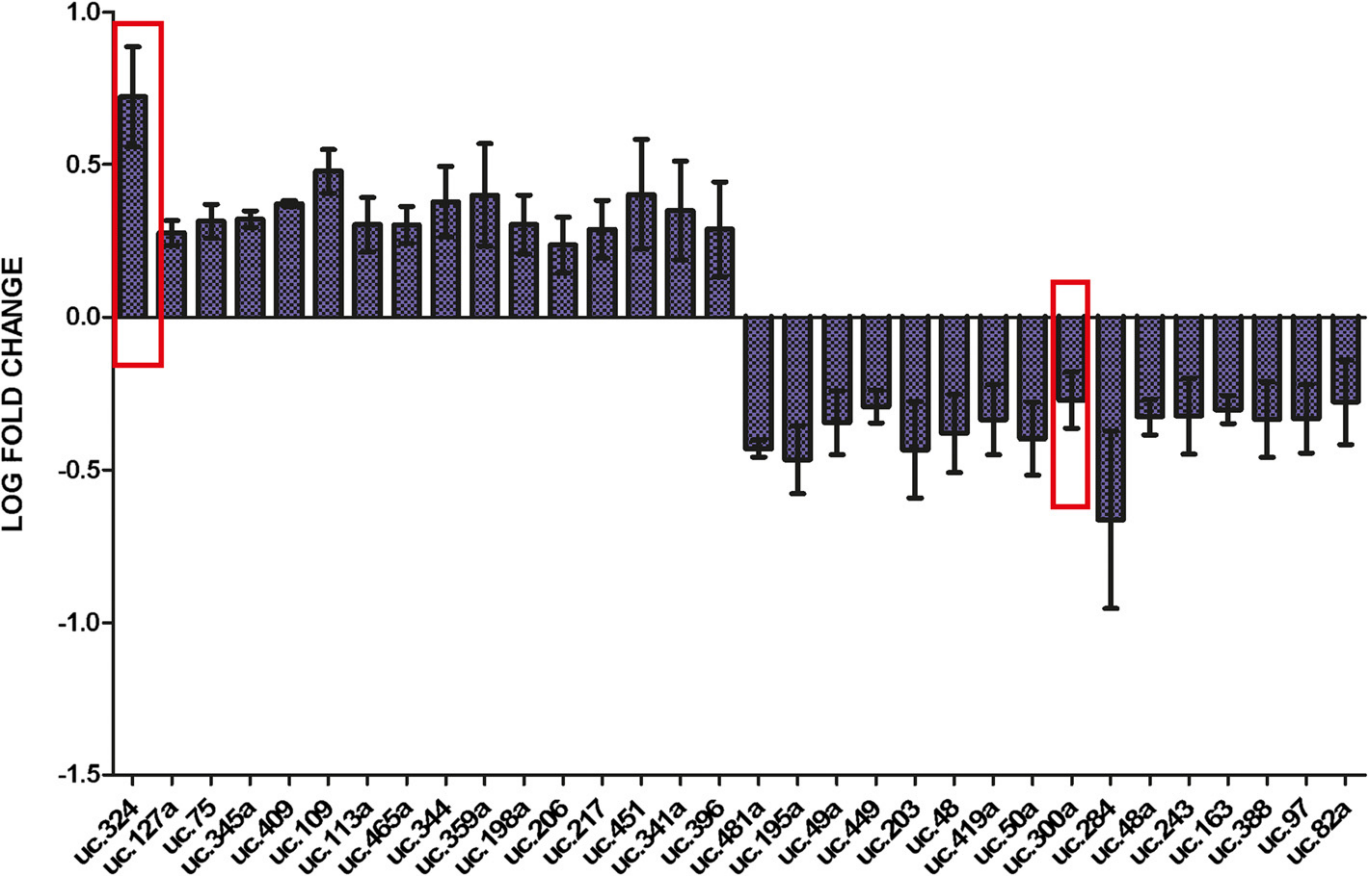
**T-UCRs expressed following ATRA-treatment.** Thirty-two T-UCRs were differentially expressed following ATRA-induced differentiation of neuroblastoma cell lines with p<0.05, with 16 transcripts up-regulated and 16 down-regulated greater than 1.5 fold in at least 4/6 samples. The red boxes indicate the two T-UCRs selected for PCR validation and functional analysis.

**Figure 2:**
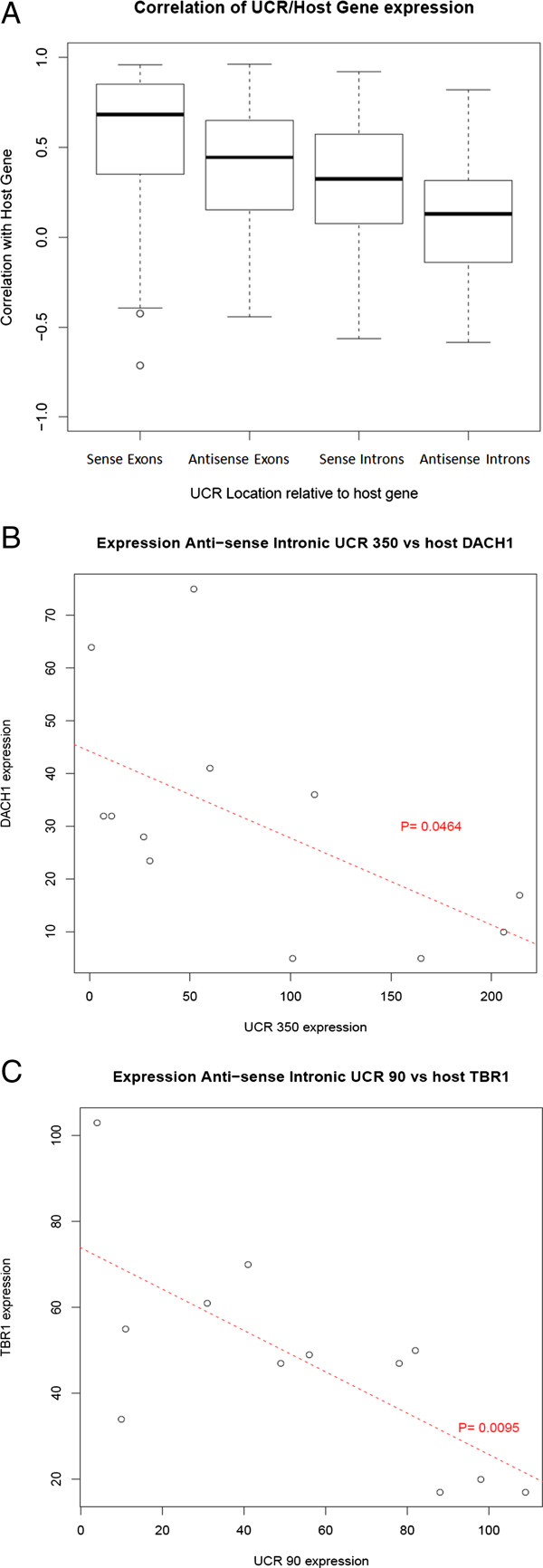
**T-UCR-host gene correlation.** Intragenic T-UCRs were assessed for correlative expression relative to host gene exonic expression, if exons were covered by the tiled region. (**A**) Box plots showing correlations between intragenic T-UCR expression and host gene expression. Anti-sense intronic T-UCRs displayed the lowest correlation with host genes, and sense exonic T-UCRs displayed the highest. The expression of anti-sense intronic T-UCRs (**B**) T-UC.350 and (**C**) T-UC.90 is significantly anti-correlated with the expression of their host genes DACH1 and TBR1 respectively (p<0.05).

In order to identify putative T-UCRs involved in neuroblastoma cell differentiation, three ATRA-sensitive NB cell lines, SK-N-BE (n=2), LAN-5 (n=2) and SH-SY5Y (n=2), were treated daily with ATRA for 7 days, which resulted in extensive neurite outgrowth. Differentiation was also confirmed at protein level through the observed up-regulation of the differentiation marker TUBB3 and down-regulation of MYCN (Additional file 1: Figure S1). Expression analysis using the tiling arrays identified 32 transcripts whose expression was significantly altered (p<0.05) following ATRA-induced differentiation (fold change of ≥1.5 in at least 4 out of the 6 biological repeats) (Figure 1, Table 1). The array results were validated by qPCR for selected T-UCRs using custom designed TaqMan assays (Additional file 2: Figure S2). The up-regulation of T-UC.324 was confirmed in all three cell lines. Levels of T-UC.300A could not be detected by qPCR in SK-N-BE, however its down-regulation was validated in both SH-SY5Y and LAN-5. The intergenic T-UCRs (9/32) can be considered independent transcripts as they are not embedded in protein coding host genes. Among the intragenic T-UCRs, 10 were transcribed in the opposite direction to their host gene, and therefore also represent independently transcribed ncRNAs. For the partially exonic or intronic T-UCRs that were expressed in a sense direction with their host gene, at least 3 likely represent independent transcripts as their level of expression is not significantly correlated with the host exon expression (e.g. host gene down-regulated in response to ATRA while T-UCR is up-regulated and vice versa). The correlation of T-UCR/host gene expression could only be carried out for intragenic T-UCRs that had host gene exons within their surrounding tiled regions (2500 bp upstream and 500 bp downstream of each UCR). Exonic T-UCRs expressed in the sense direction relative to their host gene had the highest correlations with their host genes (median correlation = 0.68), with 67% showing a significant correlation (p<0.05). Intronic T-UCRs expressed anti-sense to their host gene displayed the lowest correlation with their host genes (median correlation = 0.13), with just 12% showing a significant correlation (p<0.05) (Figure 2A; Additional file 3: Table S1). In summary, 22 independent non-coding transcriptional units that were responsive to ATRA could be identified, while 5 ATRA responsive intronic or exonic T-UCRs were identified whose expression was significantly correlated with their host genes. Consistent with these findings, some of the T-UCR host genes, such as HOXC4, are known to be responsive to ATRA [22].

**Table 1:** T-UCRs altered by ATRA

**T-UCR name**	**SK-N-BE (n=2)**	**LAN-5 (n=2)**	**SH-SY5Y (n=2)**	**Type**	**Orientation with host gene**	**Host gene**	**UCR_host correlation**	**Previously reported data**	**p-value**
**uc.324**	5.57	2.68	9.86	Exon	anti-sense	MPPED2			0.0019
**uc.127a**	2.02	2.12	1.57	Intergenic	0	0			0.0026
**uc.75**	1.86	2.66	1.77	Exon	anti-sense	ZEB2			0.0030
**uc.345a**	2.02	2.37	1.92	Intron	anti-sense	HOXC4			0.0066
**uc.409**	2.29	2.45	2.33	Intergenic	0	0		FRA<2MB (Calin et al., 2007)	0.0107
**uc.109**	3.58	2.16	3.52	Intron	anti-sense	LOC375295		HOX cluster (Calin et al., 2007)	0.0123
**uc.113a**	2.46	2.48	1.33	Intergenic	0	0			0.0134
**uc.465a**	2.62	1.89	1.63	Intron	anti-sense	POLA1			0.0154
**uc.344**	1.41	2.86	3.38	Intron	sense	HOXC4	Sig. correlated	FRA<2MB (Calin et al., 2007)	0.0156
**uc.359a**	5.08	2.35	1.33	Exon	sense	NOVA1	Sig. correlated		0.0194
**uc.198a**	1.31	2.25	2.76	Intergenic	0	0			0.0204
**uc.206**	1.14	2.01	2.24	Intergenic	0	0			0.0245
**uc.217**	1.25	2.39	2.44	Exon	sense	VSTM2A	Sig. correlated		0.0256
**uc.451**	1.15	3.01	4.67	Intron	anti-sense	TSHZ3			0.0297
**uc.341a**	1.28	4.54	1.92	Exon	anti-sense	HOXC10		FRA<2MB (Calin et al., 2007)	0.0303
**uc.396**	1.11	3.77	1.75	Intergenic	0	0		Down-regulated in HCC (Calin et al., 2007)	0.0346
**uc.481a**	0.38	0.41	0.33	Exon	anti-sense	STAG2			0.0005
**uc.195a**	0.50	0.38	0.21	Intron	sense	C6orf167	No data available		0.0013
**uc.49a**	0.48	0.66	0.29	Exon	sense	FAM98A	No data available	Inferred role in differentiation for UCR49 (Mestdagh et al., 2010)	0.0082
**uc.449**	0.42	0.49	0.64	Intron	sense	ZNF536	No data available		0.0086
**uc.203**	0.18	0.47	0.59	Exon	sense	QKI	Sig. correlated	LOH (Calin et al., 2007)	0.0097
**uc.48**	0.31	0.75	0.31	Exon	anti-sense	PUM2			0.0109
**uc.419a**	0.35	0.78	0.36	Exon	sense	SFRS1	Not Correlated	FRA<2MB, HPV16<2.5MB (Calin et al., 2007)	0.0125
**uc.50a**	0.35	0.68	0.27	Intron	sense	SFRS7	Sig. correlated		0.0131
**uc.300a**	0.38	0.51	0.79	Intron	anti-sense	PAX2		LOH, HOX gene, Up-regulated in CRC (Calin et al., 2007)	0.0135
**uc.284**	0.81	0.09	0.14	Intergenic	0	0			0.0138
**uc.48a**	0.37	0.59	0.48	Exon	sense	PUM2	No data available		0.0160
**uc.243**	0.59	0.27	0.67	Intron	sense	ZFHX4	Not correlated	AMPLIF (Calin et al., 2007)	0.0176
**uc.163**	0.59	0.41	0.51	Intergenic	0	0			0.0179
**uc.388**	0.28	0.75	0.47	Intron	sense	TCF12	No data available	Up-regulated in CRC (Calin et al., 2007) Down-regulated in CRC (Sana et al., 2012)	0.0202
**uc.97**	0.55	0.65	0.28	Intron	sense	HAT1	Not correlated	HOX gene (Calin et al., 2007)	0.0307
**uc.82a**	0.40	0.37	0.99	Intergenic	0	0			0.0499

Table shows the mean fold change across two samples for each cell line following ATRA treatment. 32 T-UCRs showed significantly altered expression following ATRA treatment across all samples (p<0.05).

### Investigation of regulation through transcriptional interference

Independent intragenic T-UCRs, either sense or anti-sense, could be involved in the regulation of their host genes through different types of transcriptional interference - where transcriptional elongation has a direct, *cis*-acting suppressive effect on a second transcriptional process [23]. Analysis of all intragenic T-UCRs and their host gene expression revealed seven T-UCRs whose expression was significantly anti-correlated with that of their host gene (Additional file 3: Table S1). None of the seven T-UCRs showed a consistent change in expression across cell lines following ATRA-treatment, however in individual samples T-UCR expression was significantly anti-correlated with host gene expression (p<0.05) which might suggest a form of regulation through transcriptional interference. The anti-correlation between T-UC.350 and T-UC.90 and their host genes can be seen in Figure 2B, C. Of particular interest is the T-UC.350/DACH1 anti-correlation. DACH1 is a tumor suppressor gene, whose expression is reduced in prostate and endometrial cancer correlating with increased tumor progression and invasion [24,25]. The significant anti-correlation seen here between the two transcripts (p<0.05) suggests a possible *cis* regulation between the two in different cancer types.

### siRNA knockdown and functional analysis of t-uc.300a

The prioritization of differentially expressed T-UCRs for functional assessment was based on the statistical significance of differential expression between untreated and ATRA-treated cells, in addition to other features of interest such as proximity to cancer-associated genomic regions or deregulation in other cancer types. The transcript of most interest from our list was T-UC.300A, which is down-regulated following ATRA treatment (Table 1). This UCR is located within a frequent loss of heterozygosity (LOH) region on chromosome arm 10q, anti-sense to the developmental gene *PAX2*, a gene whose under- or over-expression is associated with both pediatric and adult kidney pathology [26]. In addition, T-UC.300A levels were previously shown as up-regulated in colorectal carcinoma tumors [13]. Considering its down-regulation in ATRA-treated neuroblastoma cells, this suggests that T-UC.300A could play a role in both pediatric and adult cancers. T-UC.324 was selected as our second T-UCR for functional analysis. The most significantly up-regulated transcript across the three ATRA-treated cell lines (p=0.002) (Table 1, Figure 1), this ncRNA is expressed anti-sense to the *Mpedd2* gene, which displays an anti-tumorigenic function in differentiated NB cells and is also highly expressed [27]. Both T-UC.324 and T-UC.300A are independent transcripts, and not transcribed as part of a host gene.

**Figure 3:**
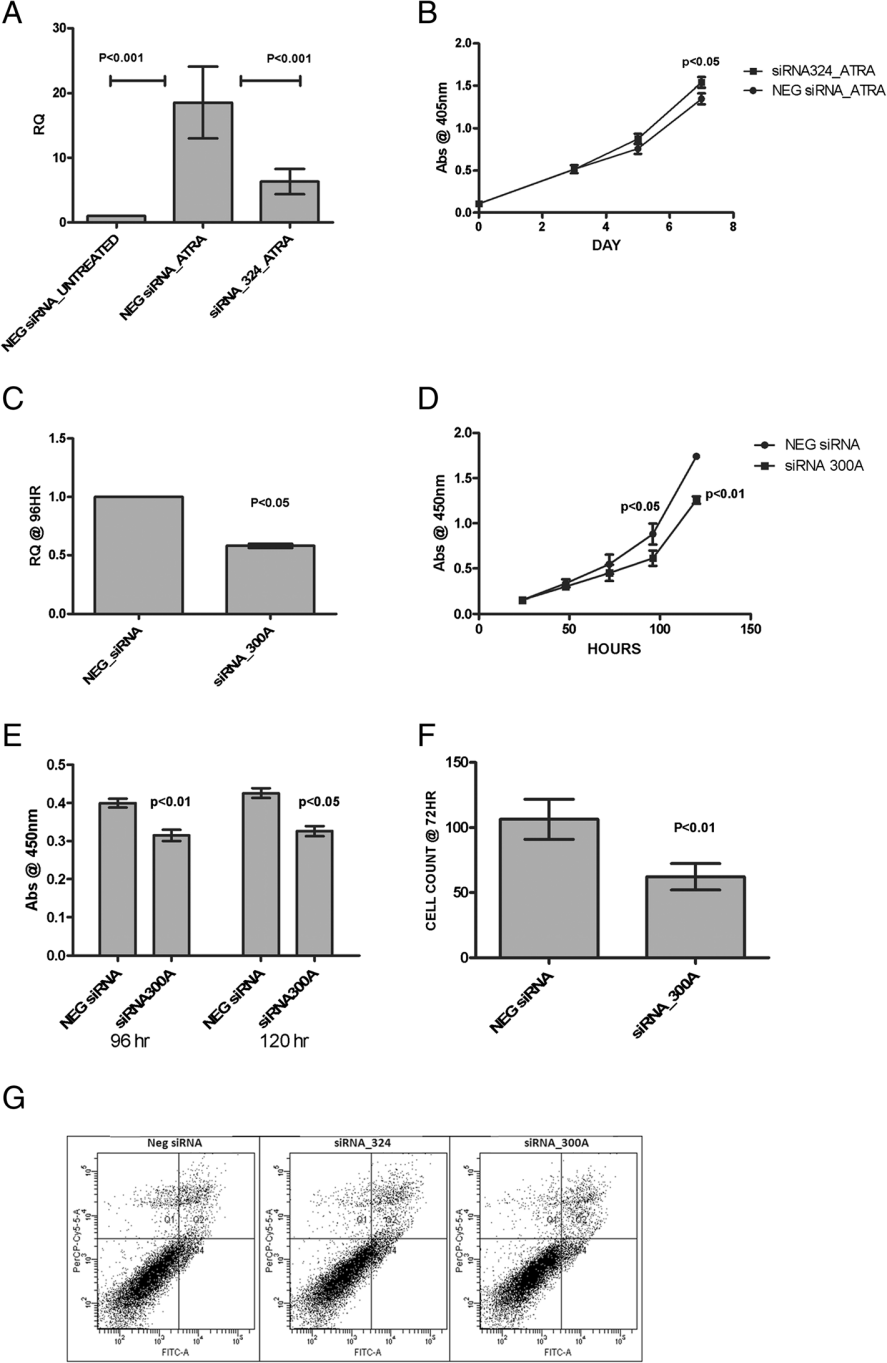
**Functional analysis of T-UCRs.** T-UC.324 and T-UC.300A transcripts were knocked down using custom designed siRNA (Dharmacon). (**A**) Knockdown of T-UC.324 significantly abrogated its up-regulation following ATRA-treatment, (**B**) but had no effect on cell viability. (**C**) A significant decrease in T-UC.300A levels was observed following siRNA transfection. (**D**) Knockdown of T-UC.300A resulted in a decrease in cell invasion as shown by MATRIGEL invasion assay (p<0.01) and (**E**) cell viability as shown by acid phosphatase assay (P<0.01). (**F**) Reduction in cell viability following T-UC.300A knockdown was shown by BrdU assay to be due to a decrease in cell proliferation (p<0.05). (**G**) Knockdown of neither T-UC.324 nor T-UC.300A had any effect on apoptosis.

SH-SY5Y and LAN-5 cells were transfected with custom designed siRNAs against either T-UC.324 or T-UC.300A to knock down these transcripts and assess any effect of this knockdown on cell invasion and proliferation rates. Knockdown of T-UC.324 abrogated its ATRA-induced up-regulation by 40% to 60% (Figure 3A) and effected a small but significant relief from the decrease in cell proliferation induced by ATRA (p<0.05) (Figure 3B). Although this effect was statistically significant across biological repeats, it was minimal and considered unlikely to contribute to any major biological role. However, knockdown of T-UC.300A resulted in a more significant decrease (p<0.01) in cell viability (Figure 3C, D). Using the Cell Proliferation ELISA, BrdU (colorimetric) kit (Roche), we determined that this decrease in cell viability was the result of a down-regulation in cell proliferation (Figure 3E), as opposed to an increase in apoptosis (Figure 3G). In addition, knockdown of the T-UC.300A transcript resulted in a decrease in cell invasion (p=0.008) (Figure 3F). There was no obvious change in cell morphology following knockdown. As T-UC.300A is significantly down-regulated following ATRA treatment, this reduction in both proliferation and invasion rates seen with siRNA knockdown supports a role for this transcript in the inhibition of differentiation, and as a promoter of proliferation and invasion.

Gene expression microarray analysis of SH-SY5Y cells following siRNA knockdown of T-UC.300A (n=2) was carried out in an effort to identify any protein-coding genes which may be involved in T-UC.300A promotion of proliferation and invasion, or inhibition of differentiation prior to ATRA treatment. Following siRNA knockdown of T-UC.300A, 150 genes were up-regulated and 123 genes were down-regulated (at least 1.5 fold in both biological repeats). The genes with the greatest mean fold change are listed in Table 2. qPCR was carried out on COL1A2 to validate array results (Additional file 4: Figure S3). By comparing the differentially expressed genes with genes we have previously shown to be altered by ATRA in SH-SH5Y cells (n=2) [19], we found that 46 out of the 150 genes up-regulated following T-UC.300A knockdown were also up-regulated following ATRA treatment (at least 1.5 fold, n=2). In addition, 23 genes down-regulated following siRNA knockdown were also down-regulated following ATRA treatment. These results suggest that the altered expression of these genes following ATRA could be the result of changing T-UC.300A levels.

**Table 2:** Top 15 up- and down-regulated protein coding genes following siRNA knockdown of T-UC.300A in SH-SY5Y cells

**Gene name**	**Mean fold change T-UC.300A siRNA knockdown**	**Mean fold change after ATRA**
INPP5D	3.72	
CALCA	3.67	40.16
LOC221442	3.24	
LOC644242	3.23	5.28
TTC9B	2.94	2.18
GDF15	2.93	5.10
USP9X	2.78	
COL1A2	2.75	
C10orf85	2.70	
FLJ4184	2.62	
HBE1	2.53	
DPF3	2.44	
LOC402199	2.43	37.41
FLJ44635	2.42	1.65
KLF2	2.38	
RAB27B	0.35	
FLJ44124	0.37	
CNTNAP5	0.07	
PAX5	0.38	
INHBA	0.39	
PCDHGA10	0.39	
TOR1AIP2	0.39	0.23
CHR4155SYT	0.39	
ZNF718	0.40	
PRTG	0.41	
HOOK1	0.41	0.38
LOC643647	0.42	0.59
LOC643523	0.42	
IPMK	0.42	
ERCC4	0.43	

Table shows fold change following knockdown across two biological repeats and also the corresponding mean fold change following ATRA if present.

### Unsupervised analysis of T-UCR expression

In order to determine if T-UCRs had sub-groupings with characteristic expression behaviour, unsupervised analysis was performed on the intragenic T-UCR cell line expression profiles. The T-UCR data was pre-processed as described in the Methods section.

**Figure 4:**
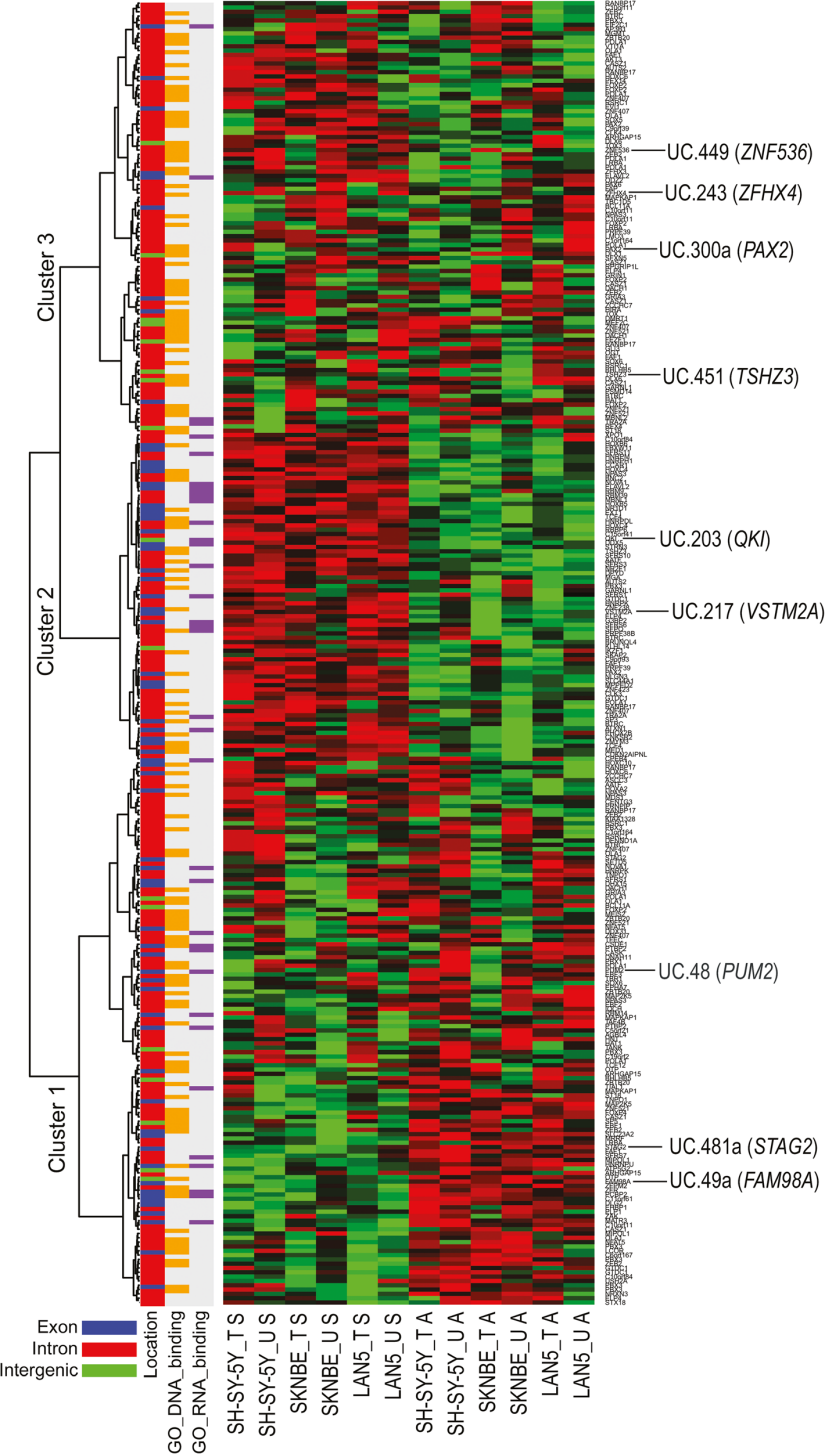
**Unsupervised analysis of intragenic T-UCR cell line expression profiles.** Subgroups of T-UCRs are expressed in three main clusters: Cluster 1, T-UCRs that are expressed anti-sense to the host gene expression; Cluster 2, T-UCRs that are expressed sense to host gene expression; Cluster 3, T-UCRs that are expressed both sense and anti-sense to host gene expression and show a significant over-representation of DNA binding proteins within their host genes relative to the other two clusters. Intragenic T-UCRs that are altered by ATRA are indicated with their host gene on the right side of the heatmap.

From the heatmap in Figure 4, we see that sub-groups of intragenic T-UCRs are expressed in three main clusters. Cluster 1 and Cluster 2 represent T-UCRs that are expressed in the anti-sense and sense direction, respectively, relative to the host gene transcription in a manner largely independent of cell line or treatment. Another subset (Cluster 3) is expressed in a more variable fashion over samples and treatments. This latter subset is significantly enriched for DNA binding proteins (*P*=2.9e-02) and has a significant under-representation of both RNA binding proteins (*P*=6.6e-04) and of exonic T-UCRs (*P*=6.0e-06). This suggests that certain sub-groups (present in Clusters 1 and 2) T-UCRs may be transcribed exclusively in a either a sense or anti-sense direction relative to the host gene whereas another subset T-UCRs (present in Cluster 3) may be transcribed in both directions relative to host gene.

## Discussion

This study represents the first profiling and functional analysis of transcribed long ultra-conserved ncRNAs that are differentially expressed during ATRA induced differentiation of NB cell lines. Although their 100% homology between human, mouse and rat denotes possible critical roles in vertebrate development, there has been limited investigation of their roles in cancer development [13-15,28,29]. Here we sought to identify and investigate the functionality of T-UCRs differentially expressed in NB cell lines following ATRA-treatment. We have identified a role for T-UC.300A in the promotion of proliferation and invasion of NB cells prior to ATRA-induced differentiation. This intronic transcript is anti-sense to the developmental transcription factor PAX2, a gene whose function is essential during embryonic development and morphogenesis but whose overexpression also contributes to the pathogenesis of diseases such as renal cell carcinoma and medulloblastoma [30,31]. There is an emerging role for ncRNAs in the regulation of HOX genes both *in cis,* through the process of transcription itself, and *in trans* through a direct role in gene regulation such as chromatin re-modelling [32,33]*.* The repressive role of T-UC.300A was alleviated by siRNA knockdown arguing against a role for transcriptional interference and, indicating that this ncRNA most likely exerts its effect *in trans*. PAX2 expression was not detected in either ATRA-treated or untreated cells, also supporting a *trans*-acting role for T-UC.300A.

Gene expression microarray analysis showed that 150 genes were up-regulated following T-UC.300A knockdown, 46 of which were also up-regulated by ATRA treatment. The gene with the greatest increase in expression, *INPP5D*, encodes the SH2-containing inositol phosphatase 1 (SHIP1), a negative regulator of the PI3K/AKT pathway. Gene transfer of *INPP5D* has been shown to reduce proliferation in CD34+ cells from patients with acute myeloid leukemia (AML) [34]. A number of the commonly differentially expressed genes are of particular interest in identifying a putative pathway for T-UC.300A function. At least two of the most highly up-regulated genes (CALCA, KLF2) have been reported as hypermethylated in a number of cancers, including neuroblastoma [35-38]. In addition, KLF2 is involved in the inhibition of proliferation and migration and is silenced in cancer by the Polycomb repressive complex 2 protein EZH2 [39,40]. Furthermore KLF2 expression in acute promyelocytic leukemia (APL) can be restored by ATRA treatment [41]. A recent study has shown that overexpression of the long ncRNA HOTAIR altered PRC2 occupancy in breast cancer cells [42]. With this in mind, it is possible that the corresponding up-regulation of genes such as KLF2, CALCA and COL1A2 following knockdown of T-UC.300A could be indicative of a role for this T-UCR in chromatin re-programming possibly by targeting methylation–associated complexes such as PRC2. Therefore reduced T-UC.300A levels (either by siRNA or ATRA treatment) could co-ordinate the de-repression of these genes by reducing methylation at their promoters and explain the observed increase in gene expression.

Our results show that the expression of a number of T-UCRs is altered by ATRA-induced differentiation. However it is clear that a number of mechanisms are at work in their regulation, and consequently in the downstream regulation of T-UCR targets themselves.

## Competing interests

The authors declare that they have no competing interests.

## Authors’ contributions

KMW carried out laboratory work pertaining to this study, participated in its design and drafted the final manuscript. KB carried out bioinformatic analyses. NHF carried out laboratory work. RLS conceived the study, and participated in its design and co-ordination and helped to draft the manuscript. All authors read and approved the final manuscript.

## Pre-publication history

The pre-publication history for this paper can be accessed here:

http://www.biomedcentral.com/1471-2407/13/184/prepub

## Supplementary Material

Additional file 1: Figure S1 Untreated and ATRA-treated SK-N-BE cells. Cells were treated with ATRA (5μM final concentration) daily for 7 days. (**A**) Cells were probed using the neuronal marker βIII Tubulin (Abcam), and were incubated with the flourescein-conjugated goat anti-rabbit Alexa Flour 488 antibody (Invitrogen). (**B**) Western Blot for ßIII-Tubulin, MYCN and α-Tubulin (loading control) in untreated and ATRA-treated cells. Click here for file

Additional file 2: Figure S2 qPCR validation of tiling array results. Array results were validated for T-UC.324 and T-UC.300A by qPCR using gene-specific RT primers and custom-designed TaqMan Assays. Error bars represent standard deviation from the mean across at least two biological repeats. Click here for file

Additional file 3: Table S1 Correlation of intragenic T-UCR and host gene expression. T-UCRs are in four groups: exonic sense; exonic anti-sense; intronic sense; intronic anti-sense. Shaded cells denote significance or approaching significance. Click here for file

Additional file 4: Figure S3 qPCR validation of gene expression results. Gene expression microarray results were validated by qPCR for COL1A2 in SHSY5Y cells following knockdown of T-UC.300A. Figure shows results from array and from qPCR analysis. Error bars represent standard deviation from the mean across two biological repeats. Click here for file
