# Supplementary figures and images for: Expressional alterations in functional ultra-conserved non-coding rnas in response to all-trans retinoic acid - induced differentiation in neuroblastoma cells
Source: BMC Cancer. 2013 Apr 8;13:184. doi: 10.1186/1471-2407-13-184 (PMC3626850; doi:10.1186/1471-2407-13-184)

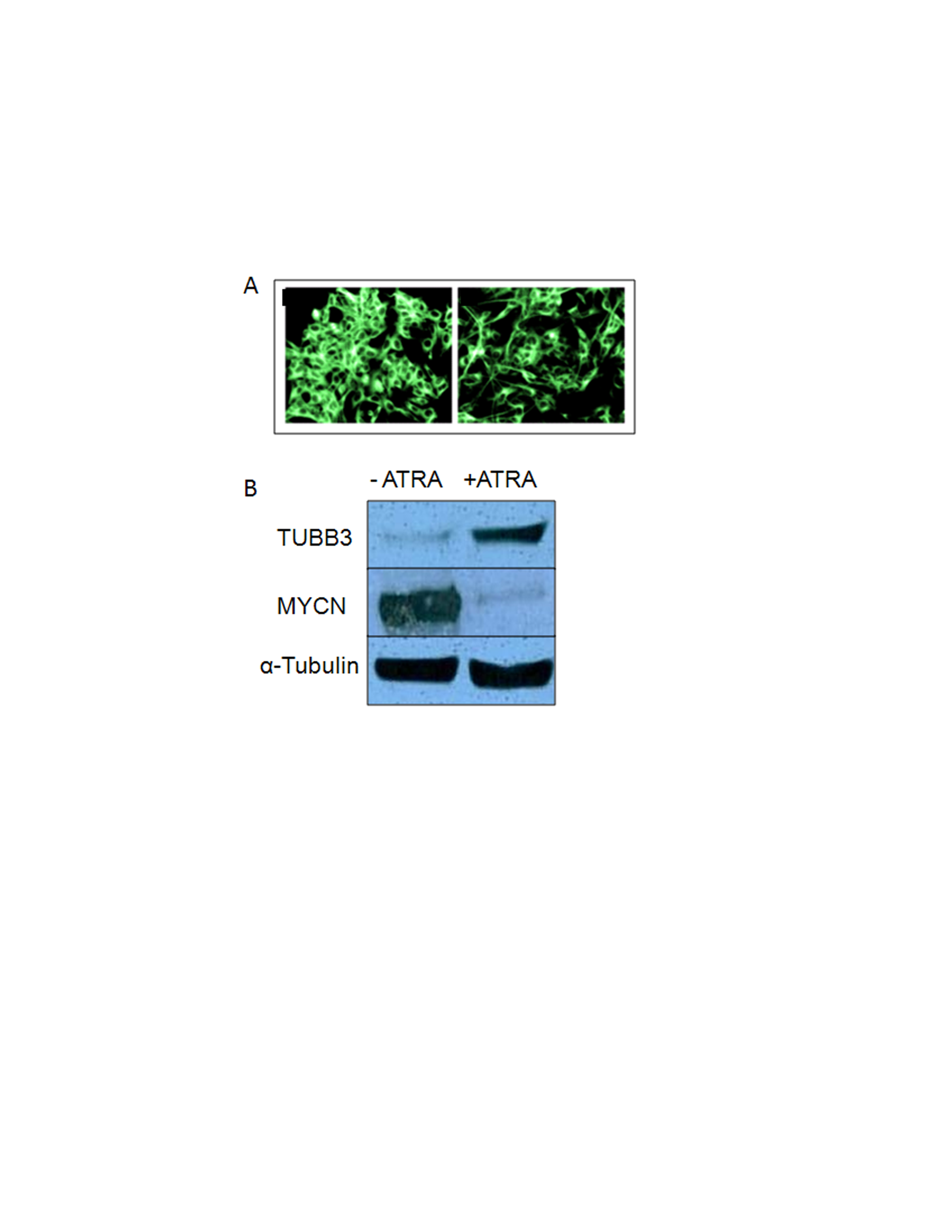

Supplement: Additional file 1: Figure S1 — Untreated and ATRA-treated SK-N-BE cells. Cells were treated with ATRA (5μM final concentration) daily for 7 days. (A) Cells were probed using the neuronal marker βIII Tubulin (Abcam), and were incubated with the flourescein-conjugated goat anti-rabbit Alexa Flour 488 antibody (Invitrogen). (B) Western Blot for ßIII-Tubulin, MYCN and α-Tubulin (loading control) in untreated and ATRA-treated cells. [file 1471-2407-13-184-S1.tiff]

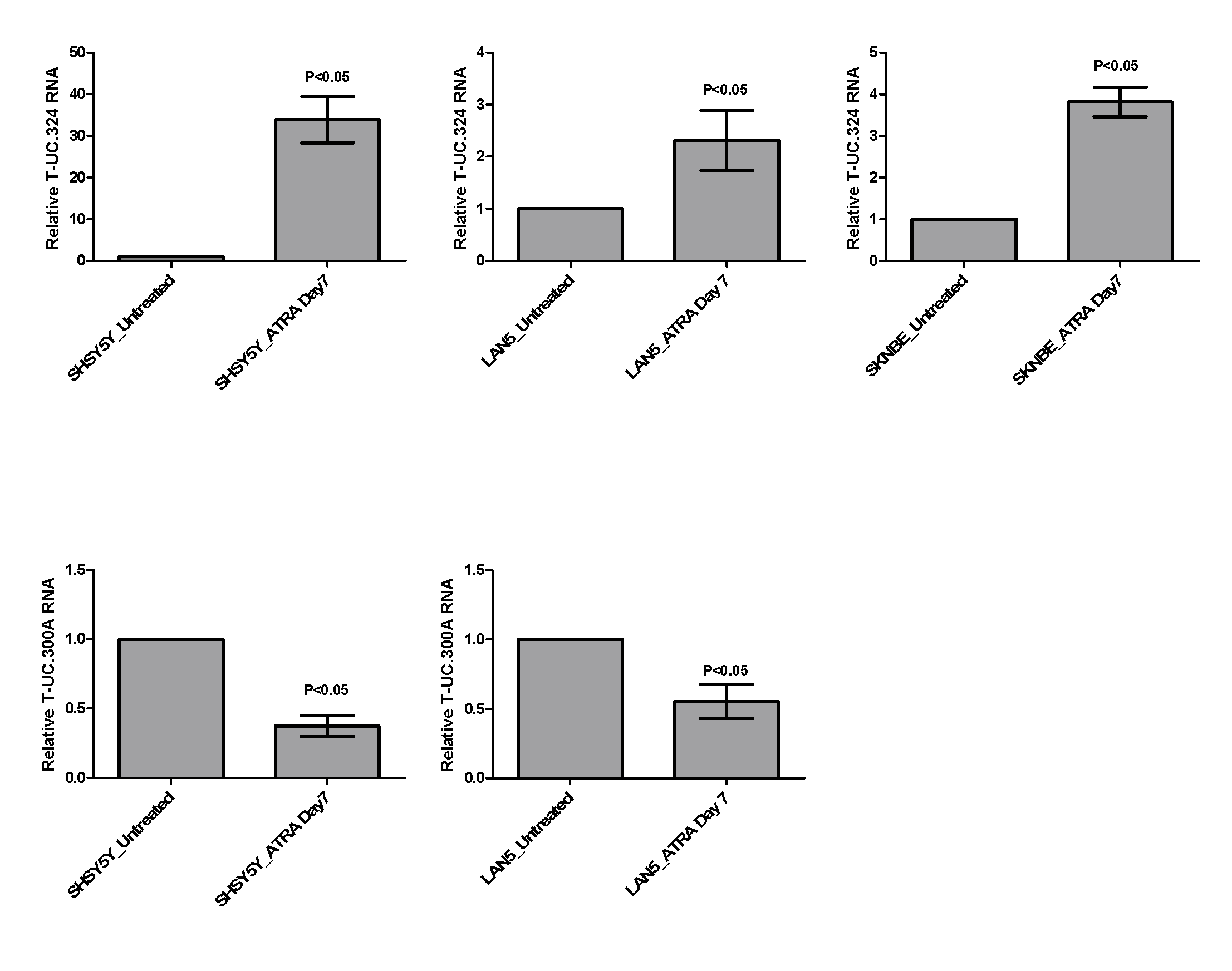

Supplement: Additional file 2: Figure S2 — qPCR validation of tiling array results. Array results were validated for T-UC.324 and T-UC.300A by qPCR using gene-specific RT primers and custom-designed TaqMan Assays. Error bars represent standard deviation from the mean across at least two biological repeats. [file 1471-2407-13-184-S2.tiff]

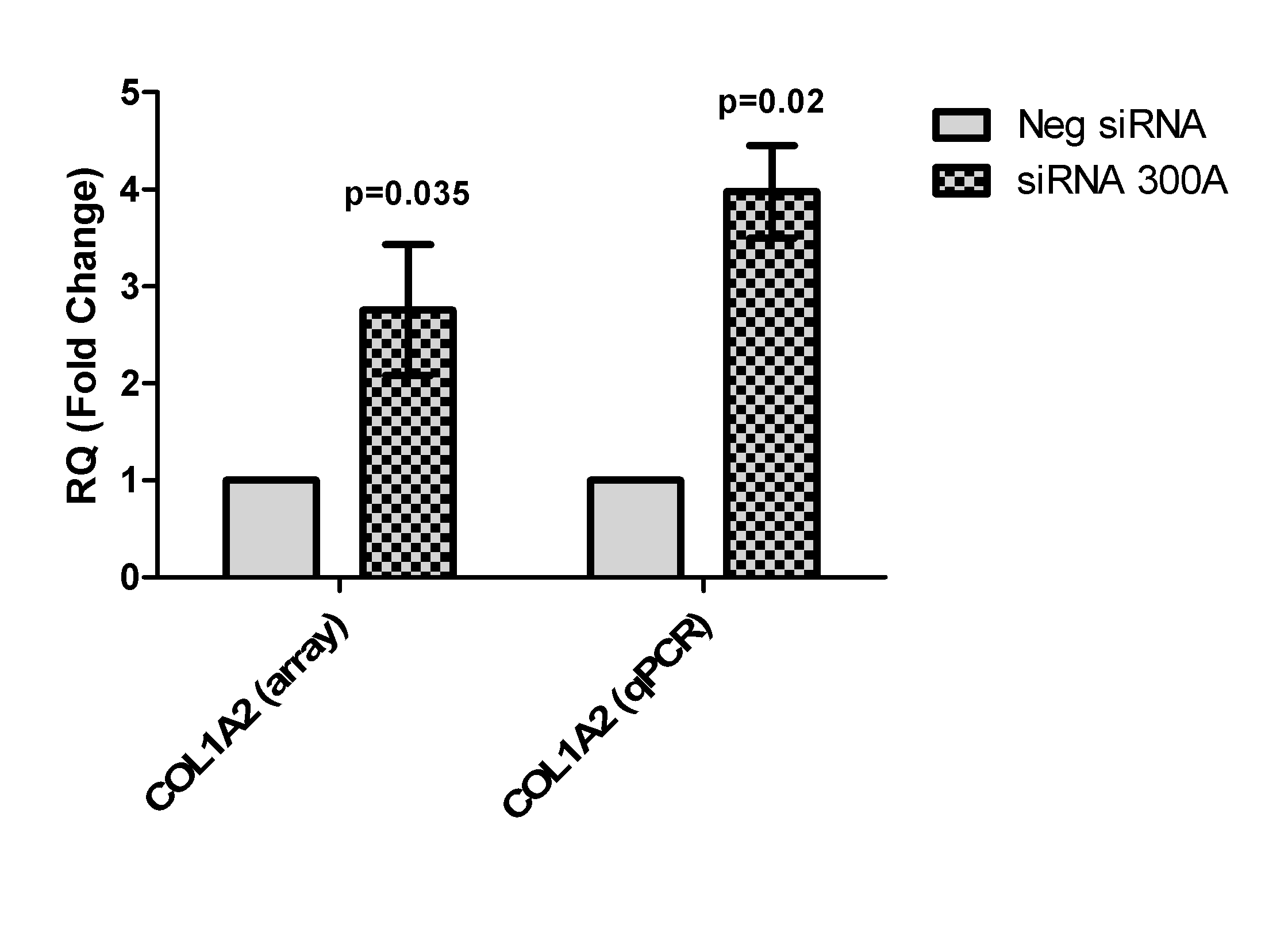

Supplement: Additional file 4: Figure S3 — qPCR validation of gene expression results. Gene expression microarray results were validated by qPCR for COL1A2 in SHSY5Y cells following knockdown of T-UC.300A. Figure shows results from array and from qPCR analysis. Error bars represent standard deviation from the mean across two biological repeats. [file 1471-2407-13-184-S4.tiff]
